# Prospective Study of Prolonged Grief Disorder in Relatives of COVID-19 Deceased

**DOI:** 10.1192/j.eurpsy.2023.1252

**Published:** 2023-07-19

**Authors:** S. N. Martins, R. Salgado, Â. Nogueira, I. Guedes, N. Carvalho, Â. Ribeiro, B. Ribeiro, D. Mendes

**Affiliations:** Centro Hospitalar Tâmega e Sousa, Penafiel, Portugal

## Abstract

**Introduction:**

COVID-19 pandemic along with its social restrictions changed our burial practices and the way we bury our dead. In consequence, it affected people’s experiences and traditions which could lead to severe, persistent, or disabling grief.

Thereby, it is relevant to understand how someone may be more susceptible to developing pathological grief and what can we do to prevent it.

**Objectives:**

To assess the risk of Prolonged Grief Disorder (PGD) in family members of patients who died from COVID-19 infection and identify possible risk factors.

**Methods:**

Prospectively, we performed follow-up interviews conducted with family members of all patients who died from COVID-19 infection in 2020 at our hospital. The sample was characterized, and clinical follow-up was performed for at least 6 months after the date of death. By that time, the PG-13 scale was applied.

**Results:**

A total of 269 individuals who had some type of relationship with patients who died from COVID-19 were included, with 68% being female with a mean age of 53.7 years.

After clinical follow-up, 10.8% of the patients met the diagnostic criteria for PGD.

Regarding the degree of kinship, the only predictor of PGD was “spouse” (OR 11,236, [4,762; 26,316]; p < 0,001). A closer and more regular interaction with the deceased was also associated with an increase in PDG (OR 5.682, [1.314; 24.390] p = 0.009).

Feelings of denial and guilt by the time of death notification were also risk predictors for PGD (OR 2,412, [1,091; 5,332] p = 0,026) and OR 2,888, [1,244; 6,703] p = 0,011, respectively).

The impossibility of being present at the funeral was associated with a risk of about 3 times higher of developing PGD (OR 3,817 [1,727; 8,403] p < 0,001).

Older age (p<0,001) and lower educational qualification (p=0,003) were also presented as risk factors.

Other characteristics including gender, marital status, previous suicide attempts, psychiatric or consumption history, previous significant bereavement or the social and family support of the person contacted were not predictors of PGD.

**Conclusions:**

The present prospective study made it possible to reinforce and support the way in which the COVID-19 pandemic, associated with significant social modifications, changed the way people experience grief.

**Disclosure of Interest:**

None Declared

